# Emerging and reemerging arboviruses: A new threat in Eastern Peru

**DOI:** 10.1371/journal.pone.0187897

**Published:** 2017-11-14

**Authors:** Carlos Alva-Urcia, Miguel Angel Aguilar-Luis, Carlos Palomares-Reyes, Wilmer Silva-Caso, Luis Suarez-Ognio, Pablo Weilg, Carlos Manrique, Fernando Vasquez-Achaya, Luis J. del Valle, Juana del Valle-Mendoza

**Affiliations:** 1 School of Medicine, Faculty of Health Sciences, Universidad Peruana de Ciencias Aplicadas, Lima, Peru; 2 Research and Innovation Center of the Faculty of Health Sciences, Universidad Peruana de Ciencias Aplicadas, Lima, Perú; 3 Laboratorio de Biología Molecular, Instituto de Investigación Nutricional (IIN), Lima, Perú; 4 Dirección Regional de Salud Madre de Dios (DIRESA-Madre de Dios), Madre de Dios, Peru; 5 Barcelona Research Center for Multiscale Science and Engineering, Departament d’Enginyeria Quıímica, EEBE, Universidad Politécnica de Catalunya (UPC), Barcelona Tech, Barcelona, Spain; CEA, FRANCE

## Abstract

**Background:**

Arboviral diseases are one of the most common causes of acute febrile illness (AFI) and a significant health problem in South America. In Peru, laboratory etiologic identification of these infections occurs in less than 50% of cases, leading to underdiagnoses of important emerging arboviruses.

**Aim:**

To assess the prevalence of the Dengue (DENV), Oropouche (OROV), Chikungunya (CHIKV), Mayaro (MAYV) and Zika (ZIKV) viruses in patients with acute febrile illness from Puerto Maldonado (Peru).

**Methodology:**

Serum samples were obtained from patients with AFI during January 2016 to March 2016. A total of 139 specimens were analyzed for the presence of DENV, OROV, CHIKV, MAYV, and ZIKV using polymerase chain reaction (PCR).

**Results:**

CHIKV in 9.4% and OROV in 8.6% were the most prevalent arboviruses, followed by DENV and ZIKV, with a prevalence of 6.5% and 5%, respectively. Among all patients, the most common symptoms accompanying fever were headaches 79.9%, muscle pain 65.5% and joint pain 63.3%.

**Conclusions:**

During this short 3-month period, 4 arboviruses were detected by PCR, CHIKV and OROV being the most common arboviruses in Puerto Maldonado (Peru). Thus, it is crucial to include OROV detection in the national health surveillance. Furthermore, the etiologic clinical diagnosis of arboviral infections is not possible due to the low specificity of symptoms; therefore an increase of cases confirmed by molecular diagnostic methods will enhance arboviral surveillance in Peru.

## Introduction

Arthropod-borne viruses, also known as Arboviruses are one of the most common etiologic agents responsible for human febrile illness worldwide and an emerging concern in recent decades [1]. Arboviruses have a high mutation and adaptation capacity, which gives them the ability to cause outbreaks. Recently these infections have become a significant health problem in South America [2,3], especially in tropical regions, where high temperatures and humidity favor mosquito proliferation and therefore the transmission of arboviruses [1,3].

Acute febrile illness (AFI) is defined as fever of less than a 1 week of duration, without an identified source of infection that may be accompanied by other symptoms. Many infectious agents have been responsible for this syndrome, including arboviruses. However, the infectious etiology and epidemiology of AFI, remains poorly characterized, especially in low and middle-income countries, where laboratory analysis is hindered by limited resources [1,4,5].

In South America, dengue and leptospirosis are described as the most predominant cause of AFI [5]. However, in the Amazon Basin, dengue viral infections represent around 6–26% of patients with AFI, and in dengue-endemic areas, co-circulating pathogens are often misclassified [1]. Moreover, in low to middle-income countries, an overdiagnosis and overtreatment of malaria have been recentlyreported [5,6].

Molecular techniques such as polymerase chain reaction (PCR) have broadened the spectrum of AFI etiologies and emphasized the role of Arthropod-borne viruses, present in up to 32.5% of all febrile cases [1,5]. Arboviruses are a heterogeneous group; however, the most relevant belong to few viral genera including *Flavivirus*, e.g. Dengue virus (DENV) and Zika virus (ZIKV); *Alphavirus*, e.g. Chikungunya virus (CHIKV) and Mayaro virus (MAYV); and *Orthobunyavirus*, e.g. one of the most common is the Oropouche virus (OROV) [1–3].

In Peru, a rapid increase of dengue outbreaks was observed in 2015, with 39,440 of cases reported, double the number in comparison to the previous year (17,234 cases at 2014) [7]. The most affected regions with 57.4% of cases registered were Ayacucho, Cuzco, Piura and Madre de Dios [8]. However, despite the efforts by the national health surveillance program to monitor dengue in 2015, only 49.2% of cases were laboratory-confirmed, and among these, there were reports of etiologic misdiagnosis. [6,8].

On February 2016, the World Health Organization (WHO) declared the Zika virus (ZIKV) an epidemic in Brazil and a public health emergency of international concern due to its associated neurological complications in newborns [9]. Since 2015, ZIKV has become an increasing threat to Peru as four neighboring countries have reported endemic cases of this disease. [8,10,11]. However, until January 2016, the national health report has not identified any cases of ZIKV since its epidemiological alert was issued in October 2015 [12].

On the other hand, Chikungunya (CHIKV) alphavirus has also been recognized as an important cause of acute febrile illness in recent years after it was first identified in the South America late 2013. In 2015, this virus showed a high frequency of 49.5% of the 208 endemic cases reported [6]; but unfortunately, a low confirmation rate was reported for this high incidence. The Mayaro (MAYV) alphavirus is also being investigated in Peru due to small outbreaks reported in the last 15 years [13], and is also being considered as a possible etiologic agent of AFI especially when during dengue outbreaks [14]. A study in the Amazon basin region of Peru during the period 2010–2013 reported for MAYV a prevalence of 0.8% (16/2094) in febrile patients [15].

Finally, Oropouche virus (OROV) is an under-reported etiologic agent of AFI, which causes symptoms similar to those of dengue and has been detected in mixed outbreaks of dengue in Madre de Dios (Peru) [16]. In 2011, a study in Iquitos, located in the Peruvian Amazon, reported that OROV was responsible for 14.9% of acute febrile infections [17]. Additionally, in January 2016, a study from Madre de Dios in Peru, analyzed samples that were previously negative for DENV and found OROV in 24% of cases; to date, this result has been the most significant OROV outbreak in Peru with 120 confirmed cases [18]. Due of these outbreaks, OROV has been identified as a potential cause for epidemics and co-infections and the WHO has advised Peru to focus on preventive measures for vector control and surveillance [16].

To study the arboviral epidemiology in Peru is essential to understand their real impact in our community. This study aimed to detect, through RT-PCR, the presence of DENV, CHIKV, ZIKV, MAYV, and OROV in samples of patients with Acute Febrile Illness (AFI) from Puerto Maldonado (Peru) and to assess preliminary epidemiological and clinical data of these patients.

## Materials and methods

### Patients and sampling

A consecutive cross-sectional study was conducted in Puerto Maldonado in coordination with the *“Dirección Regional de Salud Madre de Dios*.” Puerto Maldonado is the capital of the Madre de Dios Region, a city in Southeastern Peru in the Amazon rainforest 55 km West of the Bolivian border. Puerto Maldonado has an estimated population of 74,949 and is recognized as an endemic area for dengue and other arboviruses [7]. Madre de Dios was chosen as the area of study as it is one of the most affected regions with DENV in Peru; it is located next to Brazil and Bolivia, where native cases Zika virus have been previously reported and it is also where the most significant outbreak of OROV was reported in 2016 [8, 9].

Patients that fulfilled the selection criteria were studied from January to March 2016. The inclusion criteria were patients who arrived to Internal Medicine-Pediatrics outpatient clinics with acute febrile illness (greater than or equal to 38°C axillary temperature in the previous 7 days) along with one or more of the following symptoms: headache, muscle pain, retro-ocular pain, joint pain, nausea, low appetite, vomiting, dizziness, abdominal pain, diarrhea, chills, rash, photophobia, sore throat, cough, pallor, rhinorrhea, dyspnea, jaundice, cough, conjunctival injection, dysuria or convulsions. Exclusion criteria included patients with an identifiable source of infection, such as sinusitis, pneumonia, acute otitis media and acute upper respiratory tract infections, among others.

Patients from the following primary health care facilities were included in the study: Centro de Salud Nuevo Milenio, Centro de Salud Santa Rosa, Centro de Salud Laberinto, Centro de Salud La Joya, Centro de Salud Bélgica, Centro de Salud Iñapari, Centro de Salud Jorge Chávez, Centro de Salud El Triunfo and Centro de Salud Tres Islas.

### Ethics statement

This study was approved by the Research Ethics Board of the *Hospital Regional de Cajamarca*, Peru. A written informed consent was signed before enrollment; for participants under 18 years old the informed consent was signed by parents or children caregivers before enrollment.

### Samples

One serum sample per patient was collected by using Vacuette^®^ TUBE Serum Separator Clot Activator (Vacuette, Greiner Bio-One, Kremsmünster, Austria). After collection, all the samples were stored at -80°C. All 139 samples were transported to Lima (Peru) under standardized frozen conditions to perform molecular assays. Positive control material for DENV, CHIKV, ZIKV, and MAYV was provided by the Centers for Disease Control and Prevention (CDC, Fort Collins, CO, USA).

### RNA extraction

RNA extraction was performed from 200 μL of the serum samples with the High Pure RNA Isolation Kit (Roche Applied Science, Mannheim, Germany), according to the manufacturer’s instructions. Viral RNA obtained after extraction was eluted in 100 μl of nuclease-free water and then processed or stored at -20°C until use.

### Real-time RT-PCR assay for detection DENV, CHKV and ZIKV with taqman probe

A one-step RT-PCR was performed using TaqMan with BHQ quencher probe at 125 nM and 250 nM of primers in a final volume of 20 μL. Five microliters of the extracted RNA was combined with 15 μl of the master mix and the reverse transcription step was performed 95°C for 15 minutes, 60 cycles of 15 seconds at 95°C and 45 seconds at 60°C. All the procedure was performed in Light Cycler^®^ 2.0 Instrument and data was analyzed with the LightCycler^®^ Software 4.1 (Roche Diagnostic, Deutschland-Mannheim, Germany). The primers and the probe used are shown in Table 1 [19–21].

**Table 1:** Primers and probes used in the PCR assays.

Primer	Sequence (5´-3´)	Amplicon (pb)	Ref.
DENV-F	AGG ACY AGA GGT TAG AGG AGA	107	[19].
DENV-R	CGY TCT GTG CCT GGA WTG AT		
DENV-Probe	FAM-ACA GCA TAT TGA CGC TGG GAR AGA CC-TAMRA		
CHIKV-F	AAG CTY CGC GTC CTT TAC CAA G	209	[20].
CHIKV-R	CCA AAT TGT CCY GGT CTT CCT		
CHIK-Probe	FAM-CCA ATG TCY TCM GCC TGG ACA CCT TT-TAMRA		
ZIKV-F	AAR TAC ACA TAC CAR AAC AAA GTG GT	109	[21].
ZIKV-R	TCC RCT CCC YCT YTG GTC TTG		
ZIKV-Probe	FAM-CTYAGACCAGCTGAAR-TAMRA		
OROV-F	GTG GGG TCC AAT TTG C	300	[22].
OROV-R	TGA ACC CTA TGC ATC T		
MAYV-F	TTC CRA AYC AAG TGG GAT TC	166	[23].
MAYV-R	CAC TTT ACG TAY GGK GAT GG		

### Detection MAYV and OROV by conventional PCR

For the reverse transcription (RT), a 20 μl mixture was prepared to contain 5 μl of RNA extracts, the Transcriptor High Fidelity cDNA Synthesis Kit (Roche Applied Science, Mannheim, Germany) was used according to the manufacturer’s instructions. A 166 bp and 300 bp fragments were amplified for Mayaro and Oropouche viruses respectively [22,23]. Table 1 shows the primers used for amplification. The final volume of the PCR mixture was 50 μl, distributed as follows: 25 μl of enzyme mix (Taq polymerase, 2.5 mM MgCl_2_, 15 mM Tris / HCl pH 8.3, 50 mM KCl, 200 μM of each deoxynucleotide), 20 pmol of each primer (Macrogen, Seoul, Korea), and 5 μl of DNA extraction. Thus, the PCR conditions were: 95°C for 10 min, followed by 55 cycles of 94°C for 1 min, 55°C for 1 min and 72°C for 1 min, with a final elongation of 10 min at 72°C. The amplified DNA products were analyzed by gel electrophoresis on 2% agarose (FMC, Rockland, ME) gel containing ethidium bromide (3 mg/L). Amplified products were gel recovered, purified (SpinPrep^™^ Gel DNA Kit, San Diego, USA) and sent to be sequenced (Macrogen, Seoul, Korea).

### Data analysis

Qualitative variables were reported as frequencies and percentages. All analyses were processed with the IBM Statistical Package for the Social Sciences (SPSS) software version 21.0 (SPSS, Chicago, IL, USA). One-way ANOVA followed by the Tukey test were performed for comparison between groups. The frequency distributions of clinical symptoms were analyzed using a paired sample t-Test, and the Pearson correlations were performed in a pairwise manner. A *p*-value <0.05 was considered statistically significant.

## Results

A total of 139 patients with acute febrile illness (AFI) were studied from January to March 2016. Patients were grouped by ages, and the group between 20 to 44 years old corresponding at 58.3% of cases, followed by the group between 5–19 years old with 21.6% cases, and patients older than 45 years old were at 15.8% of the cases. Furthermore, no differences were observed between genders (Table 2).

**Table 2:** Demographics in patients with arboviral infections from Puerto Maldonado, Peru.

CHARACTERISTICS	TOTAL POPULATION	RT-PCR CONFIRMED ARBOVIRUS			
		Dengue	Oropouche	Zika	Chikungunya
	n (%)	n (%)	n (%)	n (%)	n (%)
**Age**					
00–04	6 (4.3)	0 (0.0)	0 (0.0)	0 (0.0)	1 (7.7)
(5–19)	30 (21.6)	1 (11.1)	1 (8.3)	2 (28.6)	2 (15.4)
(20–44)	81 (58.3)*	5 (55.6)*	7 (58.3)*	4 (57.1)*	7 (53.8)*
(45-+)	22 (15.8)	3 (33.3)	4 (33.4)	1 (14.3)	3 (23.1)
**Gender**					
Masculino	76 (54.7)	5(55.6)	8 (66.7)	4 (57.1)	5 (38.5)
Femenino	63 (45.3)	4(44.4)	4 (33.3)	3 (42.9)	8 (61.5)
**Total**	139 (100.0)	9 (100.0)	12 (100.0)	7 (100.0)	13 (100.0)
**Positive cases**	29.5%	6.5%	8.6%	5.0%	9.4%
**CI 95%**	22.6–37.6%	3.4–11.8%	5.0–14.5%	2.5–10.0%	5.6–15.3%

* ANOVA-Tukey Test, p-value<0.05.

The molecular diagnosis of the DENV, OROV, ZIKV, CHKV, and MAYV viruses in this study allowed us to identify an etiological agent in 29.5% (CI95%: 22.6–37.6%) of all cases of clinically diagnosed AFI. Thus, CHIKV and OROV were the arboviruses identified with most prevalence in 9.4% and 8.6%, respectively. While the prevalence of DENV and ZIKV were of 6.5% and 5%, respectively (Table 2). Furthermore, no cases positives of MAYV were detected in the samples studied. However, no significant differences have been observed between the prevalence of the identified viruses. To understand this result, it can be observed that CI 95% for their respective prevalence showed very similar values (Table 2).

The adult population in the range of 20–44 years was the age group that showed the highest prevalence of positive cases of DENV, OROV, ZIKV, and CHKV with values of 55.6%, 58.3%, 57.1% and 53.8%, respectively (Table 2). These prevalences were significantly higher in comparison with the prevalence of the other age groups.

In our study population, the most common symptoms accompanying the AFI were headaches in 79.9%, muscle pain in 65.5% and joint pain in 63.3%; followed by low appetite (34.5%), retro-ocular pain (33.8%) and nausea (28.8%) (Table 3).

**Table 3:** Clinical symptoms in patients with arbovirus infection positive by PCR.

Clinical symptoms	Total populationn = 139 (%)	Dengue		Oropouche		Zika		Chikungunya *	
		Positive n = 9 (%)	Odds ratio	Positive n = 12 (%)	Odds ratio	Positive n = 7 (%)	Odds ratio	Positive n = 13 (%)	Odds ratio
Headache	111 (79.86)	4 (44.44)	0.2018	8 (66.67)	0.5045	4(57.14)	0.3363	12 (92.31)	3.0270
Muscle pain	91 (65.47)	3 (33.33)	0.2637	6 (50.00)	0.5275	3 (42.86)	0.3956	11 (84.62)	2.9011
Joint pain	88 (63.31)	3 (33.33)	0.2898	7 (58.33)	0.8114	3 (42.86)	0.4347	10 (76.92)	1.9318
Low appetite	48 (34.53)	3 (33.33)	0.9479	4 (33.33)	0.9479	3 (42.86)	1.4219	6 (46.15)	1.6250
Retroocular pain	47 (33.81)	1(11.11)	0.2447	4 (33.33)	0.9787	1 (14.29)	0.3262	6 (46.15)	1.6778
Nauseas	40 (28.78)	4 (44.44)	1.9800	3 (25.00)	0.8250	3 (42.86)	1.8563	3 (23.08)	0.7425
Vomiting	11 (7.91)	2 (22.22)	3.3247	0 (00.00)	0	0 (0.00)	0	1 (7.69)	0.9697
Dizziness	9 (6.47)	1 (20.00)	1.8056	1 (8.33)	1.3131	0 (0.00)	0	1 (7.69)	1.2037
Abdominal pain	9 (6.47)	2 (22.22)	4.1270	0 (0.00)	0	0 (0.00)	0	1 (7.69)	1.2037
Chills	9 (6.47)	1(11.11)	1.8056	3 (25.00)	7.2222	1 (14.29)	2.4074	1 (7.69)	1.2037
Rash	9 (6.47)	2 (22.22)	4.1270	0 (00.00)	0	0 (0.00)	0	2 (15.39)	2.6263
Odynophagia	8 (5.76)	1(11.11)	2.0469	0 (0.00)	0	0 (0.00)	0	1 (7.69)	1.3646
Photophobia	7 (5.04)	1(11.11)	2.3571	1 (8.33)	1.7143	1 (14.29)	3.1429	1 (7.69)	1.5714
Cough	5 (3.60)	0 (0.00)	0	0 (0.00)	0	0 (0.00)	0	0 (0.00)	0
Pallor	4 (2.88)	0 (0.00)	0	2 (25.00)	6.7500	1 (14.29)	5.6250	0 (0.00)	0
Diarrhea	4 (2.88)	0 (0.00)	0	1 (8.33)	3.0682	1 (14.29)	5.6250	0 (0.00)	0
Rhinorrhea	3 (2.16)	0 (0.00)	0	0 (0.00)	0	0 (0.00)	0	0 (0.00)	0
Dyspnea	3 (2.16)	1(11.11)	5.6667	0 (0.00)	0	0 (0.00)	0	1 (7.69)	3.7778
Conjunctival injection	3 (2.16)	1(11.11)	5.6667	0 (0.00)	0	1 (14.29)	7.5556	1 (7.69)	3.7778
Expectoration	3 (2.16)	0 (0.00)	0	0 (0.00)	0	0 (0.00)	0	0 (0.00)	0
Dysuria	1(0.72)	1(11.11)	17.2500	0 (0.00)	0	0 (0.00)	0	1 (7.69)	11.5000
Jaundice	1 (0.72)	0 (0.00)	0	1 (8.33)	12.5455	0 (0.00)	0	0 (0.00)	0
Convulsions	0 (0.00)	0 (0.00)	0	0 (0.00)	0	0 (0.00)	0	0 (0.00)	0

* CHIKV vs total population, Paired t-Test, *p*-value<0.05.

In patients with DENV positive samples, both headaches and nausea were found in 44.4%. For OROV the most common symptoms were headaches (66.7%), joint pain (58.3%) and muscle pain (50%). Among the 7 positive samples for ZIKV, headache was reported in 57.1%, followed by low appetite, nausea, joint and muscle pain in 42.9% of cases. In CHIKV positive patients, the most clinical complaints with higher frequencies were headaches (92.3%), muscle pain (84.6%), joint pain (76.9%), low appetite and retroocular pain (46.2) (Table 3). Significant differences were evidenced between the symptoms reported by patients with CHIKV infection and the AFI population.

Co-infections between DENV and other arboviruses were observed in 4 patients: OROV-DENV co-infection in 2 cases which presented headache, low appetite, nausea and muscle pain; a ZIKV-DENV co-infection in 1 case whose sole clinical presentation was fever and 1 case of CHIKV-DENV who was hospitalized due to fever, chills, dizziness, shortness of breath, nausea, vomits, low appetite, abdominal pain and extreme sensitivity to light.

## Discussion

We studied 139 patients with AFI from Puerto Maldonado, Peru; in our study population most patients were between 20 to 44 years old (58.3%), and the most frequent complaints were headaches (79.86%), muscle pain (65.47%) and joint pain (63.31%). A similar demographic distribution and clinical presentation was observed in a study conducted in South America from 2000–2007 where, in addition to fever, the most commonly reported symptoms were malaise (96.7%), headache (92.2%), chills (90.2%), myalgia (81.4%) and arthralgia (76.2%) [1].

**Fig 1:**
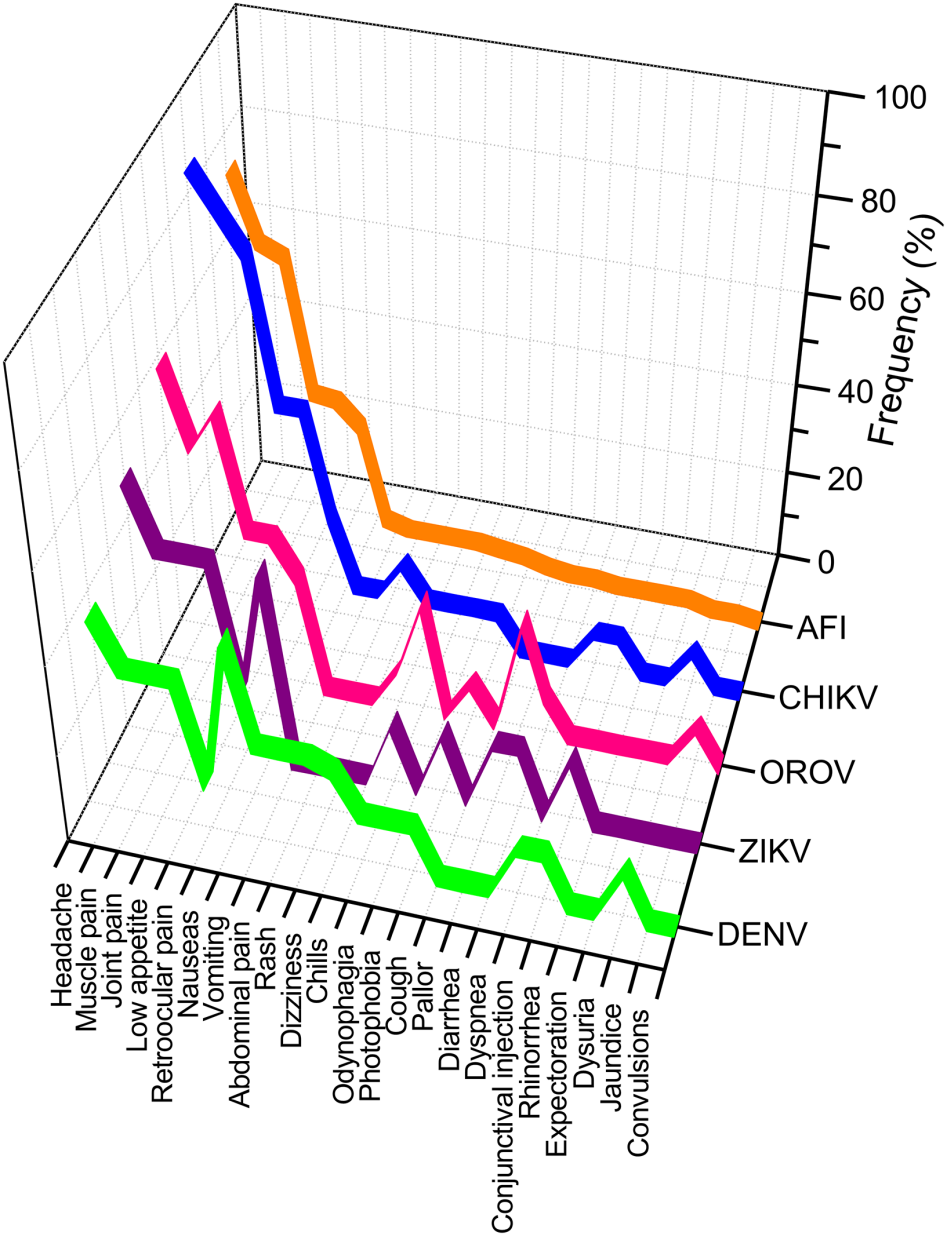
Distribution of clinical symptoms in the AFI and infections caused by arboviruses DENV, OROV, ZIKV and CHIKV.

A comprehensive analysis of the clinical symptoms frequently used for the AFI diagnosis is shown in Fig 1. Clinical symptoms for AFI were ordered by their frequency in a descending distribution and it was used a reference for comparing the clinical symptoms of DENV, ZIKV, OROV and CHIKV infections. We found that the distribution of clinical symptoms of arboviral infections correlates with the distribution of symptoms reported in cases of AFI (Fig 1). The paired analysis (*t*-Test) of the distributions has only demonstrated significant differences between AFI and CHIKV (*p* = 0.014) since in this infection the symptomatology has a higher frequency (Table 3). However, based on the distribution and frequency of symptoms it was difficult to differentiate between AFI and OROV (*p* = 0.632), ZIKV (*p* = 0.314) or DENV (*p* = 0.826).

**Fig 2:**
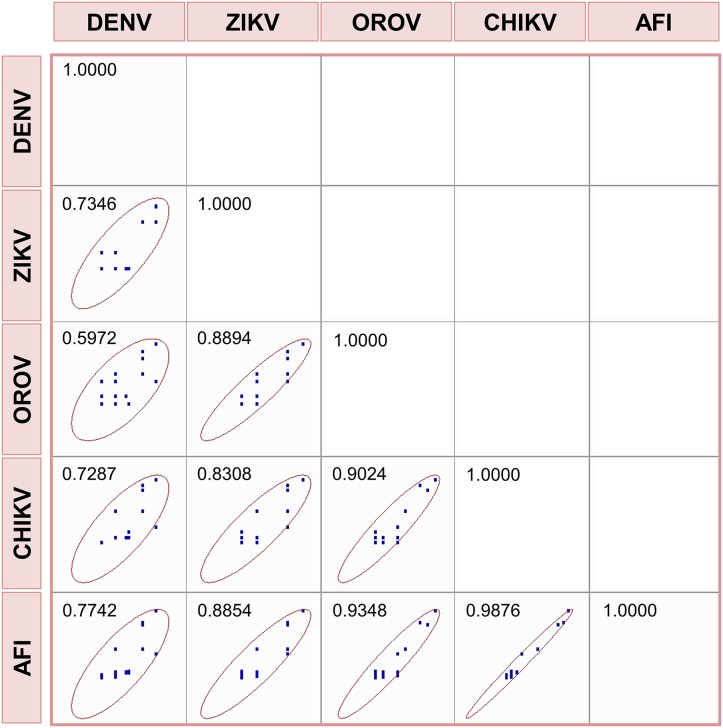
Scatter matrix and Pearson coefficient of correlation for the clinical symptoms.

In this sense, the difficulty of distinguishing arbovirus infections from AFI symptomatology is evident. Fig 2 shows the association of AFI symptoms and arbovirus infections graphically, and in all cases the associations were positive. This association was 96% and 93% for AFI and CHIKV or OROV viruses, respectively. An intermediate value of 88% was observed between AFI and ZIKV, and the lowest association of 77% was found for AFI and DENV. The main conclusion of these results is that a diagnosis of the arboviruses studied based on the symptomatology is a challenging task.

**Fig 3:**
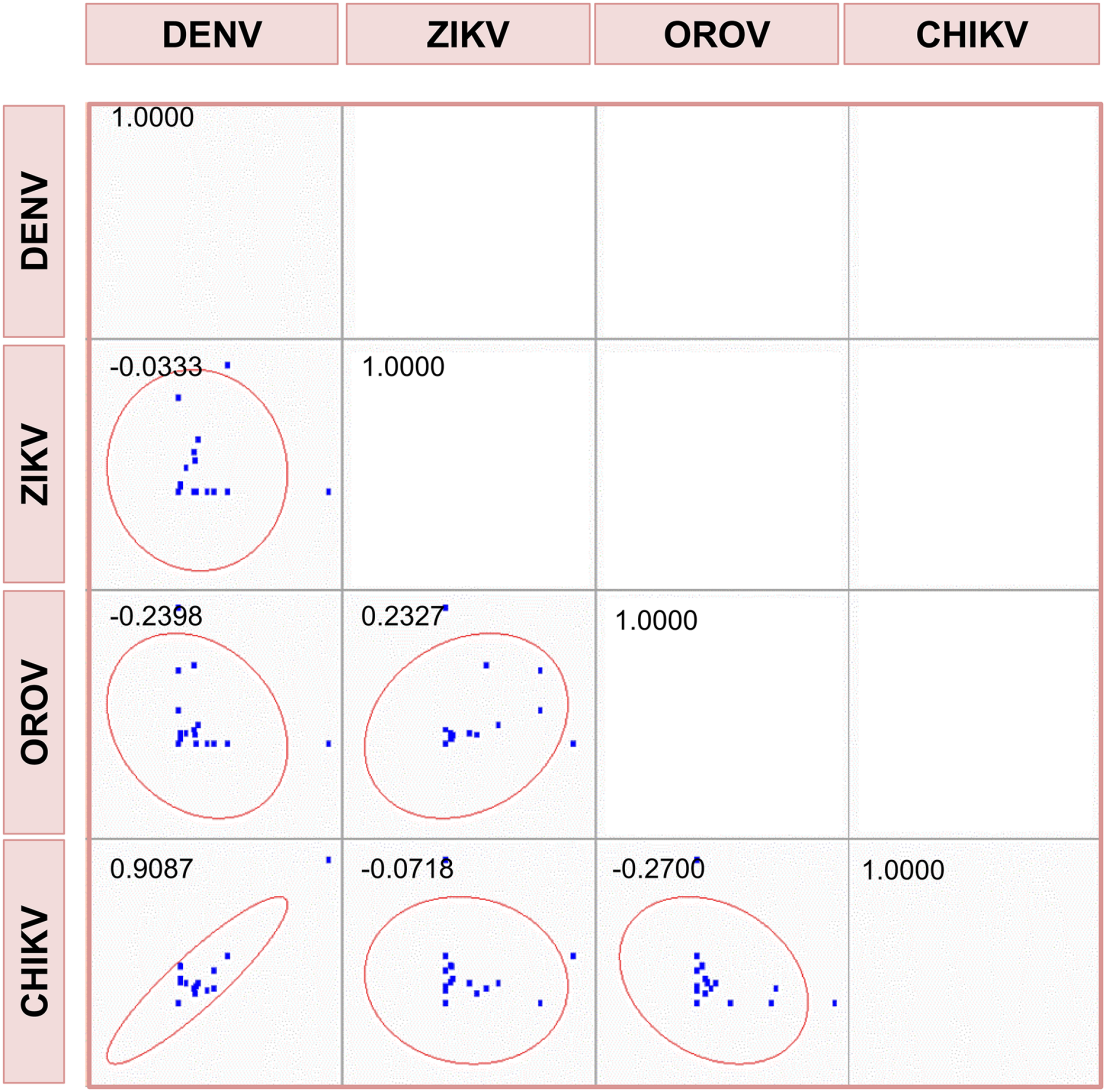
Scatter matrix and Pearson coefficient of correlation for the odd-ratios of clinical symptoms.

Based on symptom frequencies, odd-ratios were calculated for symptom control of arbovirus infections (Table 3). The association analysis of these odd-ratios shows that the odd-ratios of the symptoms of infections between DENV and CHIKV may be very similar with a correlation close to 91% (Fig 3). However, no association was found between the odd-ratios of clinical symptoms for other pairs of arboviruses (Fig 3). However, the analytical correlation of the clinical symptomatology demonstrates the enormous difficulty of making a correct and safe etiological diagnosis when arboviruses such as DENV, OROV, ZIKV or CHIKV are involved. Thus, a molecular diagnosis is essential to determine the infectious agent when these clinical symptoms so common for AFI are detected in patients.

Over the past decades, arthropod-borne viral pathogens have re-emerged in South America, especially in middle and low-income countries [1–3]. In Peru, an increase of DENV and other arboviral infections have been observed in the last 5 years [7,8]. However, only DENV, CHIKV and ZIKV are notifiable diseases in Peru, and less than 50% of samples can be confirmed by the *Instituto Nacional de Salud*, the only Laboratory in Peru designated for Epidemiological Surveillance [8,24,25]. This passive National surveillance strategy coupled with the diagnostic barriers in the rural areas have led to an underestimation of cases of arboviruses in the Peruvian Amazon Basin [1,26].

Puerto Maldonado, located in the Department of Madre de Dios in Peru, is one of the most affected areas by arboviruses; and DENV in itself accounts for a total cost of more than $200,000 dollars every year with more than 900 cases reported in 2015 [7,27]. A study in 2010, also demonstrated that Puerto Maldonado has a higher prevalence of Alphavirus infection in comparison with other regions of the Amazon Basin [1]. Furthermore, OROV has been recognized as endemic in Madre de Dios with occasional outbreaks in recent years [28,29].

In contrast to a previous study in 2010 were DENV was reported in 17.3% of patients with febrile illness in Puerto Maldonado, we only found 9 cases of DENV positive samples, representing a 6.5% of our population [1]. This lower prevalence of DENV can be related to the fact that we only used RT-PCR for case confirmation in comparison to the other study where RT-PCR and IgM seroconversion were considered as diagnostic tests. Moreover, in this previous study, a 32% of IgM cross-reactivity was observed between DENV and yellow fever virus (YFV) antigen in acute or convalescent phase samples [1]. Therefore, the limitations of implementing indirect serological diagnosis (IgM/IgG detection) should be considered in surveillance policies; specially considering that it is the most widespread test used at public health facilities [1,2].

OROV was found in 8.6% of our patients, a smaller prevalence than a previous study in Madre de Dios, were OROV was detected in 24% of samples. However, both prevalences should not be compared since the previous study was conducted analyzing DENV negative samples and again multiple laboratory tests were used (RT-PCR, ELISA IgM, and cellular culture) [17]. Moreover, it is difficult to compare our OROV prevalence with previous reports, since OROV is not a notifiable disease in Peru and recent data is unavailable [19,20]. In our study, 12 patients were positive to OROV and the most common symptoms were headaches (66.7%), joint pain (58.3%) and muscle pain (50%), which is consistent with previous studies in Iquitos and Madre de Dios where fever, headaches, joint and muscle pain were the most predominant symptoms [17,30].

In 2015, Peru reported 208 native cases of CHIKV in only 2 regions located on the northern coast: Tumbes where 91.3% of cases were observed and Piura with 8.7%. We found 13 cases of CHIKV in Puerto Maldonado including a child younger than 4 years old. Even though we cannot conclude if these 13 patients were native cases, this study is the first to report CHIKV in Puerto Maldonado.

This study is the first to report 7 cases of ZIKV in Puerto Maldonado, and this finding raises our concern since no cases have been previously reported by the Peruvian National Surveillance program until January 2016 [8]. Among the 7 patients, the most common symptom was headache (57.1%), followed by muscle pain, joint pain, nausea and low appetite (42.8%); surprisingly, none of the patients presented the classical maculopapular rash. However, due to our study design we cannot establish causality between the isolated ZIKV and the registered symptoms. Furthermore, ZIKV case definition is especially challenging during simultaneous DENV and CHIKV epidemics. [26,31]. Nonetheless, Peru is under a significant risk of a Zika and the detection of this virus in our series should raise awareness about the presence of the virus in our territories.

Our study has demonstrated a considerable number of arboviruses in Madre de Dios in a short period and a relatively small number of patients with AFI and is the first to report ZIKV in Peru. Furthermore, the high prevalence of OROV in Madre de Dios should be noted as this under-recognized virus is transmitted by biting midges and requires different sanitary measures for vector control to avoid potential outbreaks [32].

In Latin America, the main obstacles in the diagnosis of arboviral diseases are the unspecific symptoms shared by these infections at the beginning of the illness, the introduction of new pathogens and the lack of well-trained professionals to differentiate them. Therefore, health policies in Peru should focus on expanding the awareness of arboviruses among health providers and increasing the number of laboratory-confirmed cases.

We firmly believe that our National Surveillance program should be strengthened by implementing reliable diagnostic methods such as PCR that has widely demonstrated to be a fast and efficient diagnostic tool with high sensitivity for the detection of DENV, OROV, ZIKV, CHIKV, among other arboviruses [1,2,5,33]. This study should encourage further research to better understand the impact of arboviruses in the Peruvian Amazon Basin.

## References

[1] Forshey BM, Guevara C, Laguna-Torres VA, Cespedes M, Vargas J, Gianella A, et al Arboviral etiologies of acute febrile illnesses in Western South America, 2000–2007. PLoS Negl Trop Dis. 2010;4(8): e787 doi: 10.1371/journal.pntd.0000787 20706628 10.1371/journal.pntd.0000787 PMC2919378

[2] Mourão MP, Bastos Mde S, Figueiredo RM, Gimaque JB, Alves Vdo C, Saraiva Md, et al Arboviral diseases in the Western Brazilian Amazon: a perspective and analysis from a tertiary health & research center in Manaus, State of Amazonas. Rev Soc Bras Med Trop. 2015;48 Suppl 1:20–6. 26061367 10.1590/0037-8682-0133-2013

[3] Fischer M, Staples JE; Arboviral Diseases Branch, National Center for Emerging and Zoonotic Infectious Diseases, CDC. Notes from the field: chikungunya virus spreads in the Americas—Caribbean and South America, 2013–2014. MMWR Morb Mortal Wkly Rep. 2014;63(22):500–1. 24898168 PMC5779358

[4] Lorenzi OD, Gregory CJ, Santiago LM Acosta H, Galarza IE, Saint Luke's Acute Febrile Illness Investigation Team, et al Acute febrile illness surveillance in a tertiary hospital emergency department: comparison of influenza and dengue virus infections. Am J Trop Med Hyg 2013; 88:472–480. doi: 10.4269/ajtmh.12-0373 23382160 10.4269/ajtmh.12-0373 PMC3592528

[5] Iroh Tam PY, Obaro SK, Storch G. Challenges in the Etiology and Diagnosis of Acute Febrile Illness in Children in Low- and Middle-Income Countries. J Pediatric Infect Dis Soc. 2016;5(2):190–205. doi: 10.1093/jpids/piw016 27059657 10.1093/jpids/piw016 PMC7107506

[6] Naing C, Kassim AI. Scaling-up attention to nonmalaria acute undifferentiated fever. Trans R Soc Trop Med Hyg. 2012;106(6):331–2. doi: 10.1016/j.trstmh.2012.03.003 22541873 10.1016/j.trstmh.2012.03.003

[7] Red Nacional de Epidemiologia (RENACE). Casos de Dengue por Departamentos Peru 2016. [Internet]. Lima, Perú. Dirección General de Epidemiología (DGE). [Accessed on October 29, 2016; Cited on November 09, 2016] http://www.dge.gob.pe/portal/docs/vigilancia/sala/2016/SE01/dengue.pdf

[8] Ministerio de Salud del Peru (MINSA). Boletín Epidemiológico 03. [Internet]. Lima, Peru. Dirección General de Epidemiología (DGE). [Accessed November 2, 2016; Cited on November 10, 2016] http://www.dge.gob.pe/portal/docs/vigilancia/boletines/2016/03.pdf

[9] Calvet GA, Santos FB, Sequeira PC. Zika virus infection: epidemiology, clinical manifestations and diagnosis. Curr Opin Infect Dis. 2016;29(5):459–66. doi: 10.1097/QCO.0000000000000301 27496713 10.1097/QCO.0000000000000301

[10] Aguilar-León P, Bazalar-Palacios S, Rodriguez-Leyth H. The outbreak of Zika virus in the Americas: actions and challenges in Perù. Infez Med. 2016;24(2):172–3. 27367331

[11] Levy-Blitchtein S, Del Valle-Mendoza J. Zika virus is arriving at the American continent. Asian Pac J Trop Med. 2016; 9(10):1019–1021. doi: 10.1016/j.apjtm.2016.07.030 27794382 10.1016/j.apjtm.2016.07.030

[12] Ministerio de Salud (MINSA). Minsa emite alerta epidemiológica ante circulación del virus Zika en países de America del Sur. [Internet]. Lima, Perú. Dirección General de Epidemiología (DGE). [Accessed on October 29, 2016; Cited on November 11, 2016] http://www.minsa.gob.pe/?op=51&nota=17016

[13] Neumayr A., Gabriel M., Fritz J Günther S, Hatz C Schmidt-Chanasit J, et al Mayaro virus infection in traveler returning from Amazon Basin, northern Peru. Emerg Infect Dis. 2012;18(4):695–696. doi: 10.3201/eid1804.111717 22469145 10.3201/eid1804.111717 PMC3309675

[14] Zuchi N, Heinen LB, Santos MA, Pereira FC, Slhessarenko RD. Molecular detection of Mayaro virus during a dengue outbreak in the state of Mato Grosso, Central-West Brazil. Mem Inst Oswaldo Cruz. 2014;109(6):820–3. doi: 10.1590/0074-0276140108 25141284 10.1590/0074-0276140108 PMC4238776

[15] Halsey ES, Siles C, Guevara C, Vilcarromero S, Jhonston EJ, Ramal C, et al Mayaro virus infection, Amazon Basin region, Peru, 2010–2013. Emerg Infect Dis. 2013;19(11):1839–42. doi: 10.3201/eid1911.130777 24210165 10.3201/eid1911.130777 PMC3837653

[16] World Heatlh Organization (WHO). Oropouche virus disease—Peru. Disease outbreak news [Internet]. Geneva, Switzerland [Accessed on June 09, 2017; Cited on July 01, 2017] http://www.who.int/csr/don/03-june-2016-oropouche-peru/en/

[17] Aguilar PV, Barrett AD, Saeed MF, Watts DM, Russell K, Guevara C, et al Iquitos virus: a novel reassortant Orthobunyavirus associated with human illness in Peru. PLoS Negl Trop Dis. 2011;5(9):e1315 doi: 10.1371/journal.pntd.0001315 21949892 10.1371/journal.pntd.0001315 PMC3176741

[18] García MP, Merino NS, Figueroa D, Marcelo A, Tineo V E, Manrique C, et al [Detection of Oropouche viral circulation in Madre de Dios region, Peru (december 2015 to january 2016)]. Rev Peru Med Exp Salud Pública. 2016; 33(2):380–1. Spanish. 27656945

[19] Leparc-Goffart I, Baragatti M, Temmam S, Tuiskunen A, Moureau G, et al Development and validation of real-time reverse transcription-PCR for the detection and typing of dengue viruses. J Clin Virol. 2009; 45:61–66. doi: 10.1016/j.jcv.2009.02.010 19345140 10.1016/j.jcv.2009.02.010

[20] Peyrefitte CN, Pastorino BA, Bessaud M, Gravier P, Tock F, Couissinier-Paris P, et al Dengue type 3 virus, Saint Martin, 2003–2004. Emerg Infect Dis 2005;11: 757–761. doi: 10.3201/eid1105.040959 15890134 10.3201/eid1105.040959 PMC3320377

[21] Faye O, Faye O, Diallo D, Diallo M, Weidmann M, Sall AA. Quantitative real-time PCR detection of Zika virus and evaluation with field-caught mosquitoes. Virol J. 2013; 10:311 doi: 10.1186/1743-422X-10-311 24148652 10.1186/1743-422X-10-311 PMC4016539

[22] Moreli ML, Aquino VH, Cruz AC, Figueiredo LT. Diagnosis of Oropouche virus infection by RT-nested-PCR. J Med Virol. 2002;66(1):139–42. 11748670 10.1002/jmv.2122

[23] Llagonne-Barets M, Icard V, Leparc-Goffart I, Prat C, Perpoint T, André P, et al A case of Mayaro virus infection imported from French Guiana. J Clin Virol. 2016; 77:66–8. doi: 10.1016/j.jcv.2016.02.013 26921736 10.1016/j.jcv.2016.02.013

[24] Red Nacional de Epidemiologia (RENACE) Vigilancia del síndrome febril en áreas de alto riesgo de transmisión de enfermedades infeccionsas de impacto en salud publica en el Peru. [Internet]. Lima, Perú. Oficina General de Epidemiología (OGE). [Accessed on November 09, 2016; Cited on November 10, 2016] http://www.dge.gob.pe/publicaciones/pub_invepi/iepi05.pdf

[25] Ministerio de Salud (MINSA). Resolución Ministerial. [Internet]. Lima, Perú. Oficina General de Epidemiología (OGE). [Accessed on November 09, 2016; Cited on November 11, 2016] http://www.dge.gob.pe/normas/2014/RM734-2014-MINSA.pdf

[26] Tantaléan-Yépez D, Sánchez-Carbonel J, Ulloa-Urizar G, Aguilar-Luis MA, Espinoza-Morales D, Silva-Caso W, et al Arboviruses emerging in Peru: Need for early detection of febrile syndrome during El Niño episodes. Asian Pac J Trop Med. 2016;9(8):819–20. doi: 10.1016/j.apjtm.2016.06.018 27569896 10.1016/j.apjtm.2016.06.018

[27] Salmon-Mulanovich G, Blazes DL, Lescano AG, Bausch DG, Montgomery JM, Pan WK. Economic Burden of Dengue Virus Infection at the Household Level Among Residents of Puerto Maldonado, Peru. Am J Trop Med Hyg. 2015;93(4):684–90. doi: 10.4269/ajtmh.14-0755 26217040 10.4269/ajtmh.14-0755 PMC4596582

[28] García MP, Merino NS, Figueroa D, Marcelo A, Tineo V E, Manrique C, et al [Detection of Oropouche viral circulation in Madre de Dios region, Peru (december 2015 to january 2016)]. Rev Peru Med Exp Salud Publica. 2016;33(2):380–1. Spanish. 27656945

[29] Baisley KJ, Watts DM, Munstermann LE, Wilson ML. Epidemiology of endemic Oropouche virus transmission in upper Amazonian Peru. Am J Trop Med Hyg. 1998;59(5):710–6. 9840586 10.4269/ajtmh.1998.59.710

[30] Chavez R, Colan E, Phillips I. Fiebre de Oropouche en Iquitos: Reporte preliminar de 5 casos. Rev Farmacol Terap.1992;2(1):12–4

[31] Dasgupta S, Reagan-Steiner S, Goodenough D. Patterns in Zika Virus Testing and Infection, by Report of Symptoms and Pregnancy Status—United States, January 3-March 5, 2016. MMWR Morb Mortal Wkly Rep. 2016;65(15):395 doi: 10.15585/mmwr.mm6515e1 27101541 10.15585/mmwr.mm6515e1

[32] Vasconcelos P, Calisher C. Emergence of Human Arboviral Diseases in the Americas, 2000–2016. Vector Borne Zoonotic Dis. 2016 5;16(5):295–301 doi: 10.1089/vbz.2016.1952 26991057 10.1089/vbz.2016.1952

[33] Simmons M, Myers T, Guevara C, Jungkind D, Williams M, Houng HS. Development and Validation of a Quantitative, One-Step, Multiplex, Real-Time Reverse Transcriptase PCR Assay for Detection of Dengue and Chikungunya Viruses. J Clin Microbiol. 2016;54(7):1766–73. doi: 10.1128/JCM.00299-16 27098955 10.1128/JCM.00299-16 PMC4922117

